# Further Remarks on the Varioloid Disease Recently Prevalent in Derby

**Published:** 1819-06

**Authors:** Thomas Bent


THE LONDON

### Medical and Physical Journal.

6 OF VOL. XLI.] JUNE, 1819. [NO. 244.

"For many fortunate discoveries in medicine, and for the detection of nume-
"rous errors, the world is indebted to the rapid circulation of Monthly
"Journals; and there never existed any work to which the Faculty in
"EUROPE and AMERICA were under deeper obligations than to the
"Medical and Physical Journal of London, now forming a long, but an
"invaluable, series."—RUSH.

*For the London Medical and Physical Journal.*

### *Further Remarks on the Varioloid Disease recently prevalent in Derby;* by THOMAS BENT, M.D.

THE remarks of Dr. Pew on my observations on the disease which has appeared in this neighbourhood, and to which Dr. Thompson has applied the term *varioloid*, are evidently written more in the spirit of a partisan than of one intent on investigating truth. He seems to be so fully possessed with the notion of the prophylactic infallibility of cow-pox, as not to be able, for a moment, to endure its perfection being questioned. His great zeal in the cause has led him to misapprehend the tendency of my opinions.

I have not maintained that the disease was true small-pox, but, on the contrary, have distinctly stated that it differed from "*genuine small-pox;*" and the more natural inference to be deduced from the whole of my observations is, that I was rather inclined to consider the disease to be one *sui generis*. Other parts of my paper have been misinterpreted or misrepresented in a similar manner, as will be obvious to any one who will take the trouble to read it, together with Dr. Pew's remarks; and it appears, therefore, quite unnecessary for me to point them out.

I will only observe, that the cases of Mary Miller and Joseph Pegg, who had not had cow-pox, were given to illustrate the characters of the disease; which, as is distinctly observed, happened in the same form in persons vaccinated as in those who had never had cow-pox. The case of the young lady, daughter of a surgeon resident in Derby, is named in proof of this; and many others might have been given.

I mentioned formerly that the disease did not seem to be "merely small-pox modified by vaccine influence, because the symptoms were the same in those who had not been so influenced as in those who had." This has happened in frequent instances besides those above alluded to, and certainly forms a remarkable feature in the complaint; but I do not mean to maintain that it is always so, and am not sure that it is not more generally otherwise. More extended observation, and the testimony of some of my medical friends who have had much experience in the disease, incline me to think it is usually milder after vaccination than independent of it, and that it has been much more frequently fatal to those who have not previously had cow-pox.

Should this prove to be the fact, it will be one of the strongest reasons that can be given for the opinion that the disease is variolous; and the influence of cow-pox being thus made evident, proves that, although the effect of this *prophylactic* on the constitution has taken place, individuals are nevertheless liable to be attacked with small-pox. If the disease be only an aggravated form of chicken-pox, how does it happen that it is rendered milder by the influence of vaccination?

It is, perhaps, worth while to mention, by way of giving a more accurate idea of the extent to which this disease has prevailed, that it has been as general as I have ever known measles, scarlet-fever, hooping-cough, or any other contagious malady. Although recent events have lowered the value of the vaccine disease in my estimation, and I am induced to believe it less beneficial than for a time it appeared, I am still no anti-vaccinist; but, on the contrary, think mankind will be gainers by the discovery of cow-pox inoculation, and that it will appear, a milder disease has been substituted for one loathsome and dangerous.

My friend and coadjutor at the Infirmary, Dr. Forester, having expressed to me an intention of making known to you his observations and opinions on this subject very soon, and as the additional testimony of one so amply qualified cannot fail to give weight to the evidence already before the public, and as there is a near coincidence in our sentiments, I feel disinclined to extend these observations further at present.

I cannot conclude, however, without remarking, that a degree of pertness and flippancy prevails throughout Dr. Pew's observations ill-suited to the discussion of such a subject; and there is really something very ludicrous in the circumstance of an individual but little known attempting to laugh down opinions so much entitled to respect as those of Professors Monro and Thompson, and Mr. Hennen.

*Derby; April* 15, 1819.

